# Treatment outcome clustering patterns correspond to discrete asthma phenotypes in children

**DOI:** 10.1186/s40733-021-00077-x

**Published:** 2021-08-03

**Authors:** Ivana Banić, Mario Lovrić, Gerald Cuder, Roman Kern, Matija Rijavec, Peter Korošec, Mirjana Turkalj

**Affiliations:** 1 Srebrnjak Children’s Hospital, Srebrnjak 100, 10000 Zagreb, Croatia; 2 Know-Center, Infeldgasse 13, Graz, AT-8010 Austria; 3 Institute of Interactive Systems and Data Science, Graz University of Technology, Inffeldgasse 16C, AT-8010 Graz, Austria; 4 University Clinic of Respiratory and Allergic Diseases Golnik, Golnik 36, 4204 Golnik, Slovenia; 5 Faculty of Medicine, J.J, Strossmayer University of Osijek, Josipa Huttlera 4, 31000 Osijek, Croatia; 6 Catholic University of Croatia, Ilica 242, 10000 Zagreb, Croatia

**Keywords:** Machine learning, Childhood asthma, Allergy, Asthma phenotypes, Clustering, Treatment outcome

## Abstract

Despite widely and regularly used therapy asthma in children is not fully controlled. Recognizing the complexity of asthma phenotypes and endotypes imposed the concept of precision medicine in asthma treatment. By applying machine learning algorithms assessed with respect to their accuracy in predicting treatment outcome, we have successfully identified 4 distinct clusters in a pediatric asthma cohort with specific treatment outcome patterns according to changes in lung function (FEV_1_ and MEF_50_), airway inflammation (FENO) and disease control likely affected by discrete phenotypes at initial disease presentation, differing in the type and level of inflammation, age of onset, comorbidities, certain genetic and other physiologic traits. The smallest and the largest of the 4 clusters- 1 (*N* = 58) and 3 (*N* = 138) had better treatment outcomes compared to clusters 2 and 4 and were characterized by more prominent atopic markers and a predominant allelic (A allele) effect for rs37973 in the *GLCCI1* gene previously associated with positive treatment outcomes in asthmatics. These patients also had a relatively later onset of disease (6 + yrs). Clusters 2 (*N* = 87) and 4 (*N* = 64) had poorer treatment success, but varied in the type of inflammation (predominantly neutrophilic for cluster 4 and likely mixed-type for cluster 2), comorbidities (obesity for cluster 2), level of systemic inflammation (highest hsCRP for cluster 2) and platelet count (lowest for cluster 4). The results of this study emphasize the issues in asthma management due to the overgeneralized approach to the disease, not taking into account specific disease phenotypes.

**Supplementary Information:**

The online version contains supplementary material available at 10.1186/s40733-021-00077-x.

## Background

Asthma is a complex disorder of a still not completely known pathobiology, characterized by reversible airway obstruction, airway hyperresponsiveness to specific and non-specific stimuli, and a chronic inflammation in the airways. This, along with the variability in disease etiology, type and level of inflammation, bronchial damage and lung function impairment, specific clinical features and natural course of the disease (persisting to adulthood or remission in adolescence), reflects the vast heterogeneity and complexity of asthma [1]. Current knowledge of asthma pathophysiological mechanisms as a Th2 cell mediated allergic reaction does not suffice in explaining and dealing with a large portion of this heterogeneity, which is why in the past years the concept of asthma as a single disease has been revisited and redefined as a complex syndrome or an “umbrella” term encompassing several different subtypes (phenotypes) defined by newly conceived immuno-pathophysiological mechanisms- endotypes [2]. This complexity is multiplied by the fact that certain children with asthma seem to retain a specific sum of clinical features during the course of their disease, while others are known to transition to another (or several) phenotype.

A number of studies have attempted to perform asthma phenotyping by the use of unsupervised machine learning techniques. Most of them have identified age of onset- early onset vs late onset disease presentation [3–7]; gender [8]; atopy status [3, 9], obesity [5, 6] and type of inflammation- eosinophil, neutrophil, mixed type, Th2 high/low [4, 8, 10, 11] as main discriminants in distinguishing specific clusters (phenotypes). Although these studies identified several distinct phenotypes, the vast disease heterogeneity has still most likely been a major hindrance in the development of targeted therapies in asthma so far [12].

Today, common asthma treatment is actually symptomatic treatment, with short-term medications that are mostly used to relieve current symptoms and long-term medications used in case of persistent symptoms to control the underlying inflammation and prevent exacerbations. There is a marked patient-to-patient variability as well as intra-individual repeatability in the therapeutic response for all common medication classes in asthma management, indicating that the level of treatment response in asthma might have a strong genetic basis. A significant proportion of children with asthma have poor (partial or none) response when using currently available anti-inflammatory drugs [13]. Although asthma cannot be cured, with appropriate management adequate control and good quality of life can be achieved [14]. Still, even the latest GINA guidelines and recommendations, involving symptom control and exacerbation risk do not offer adequate insight into disease etiology and true level of asthma control. Also, there are no recommendations as to treatment failure identification and changes recommended towards the treatment of choice (different drug classes or their combinations) or only general choice recommendations are made (the physician can choose between several treatment options with the generally preferred option recommended). Moreover, few phenotyping studies to date have focused on treatment success as a study outcome despite the evident issues in treatment efficacy in asthma [9, 15].

In this study we attempted to utilize hierarchical clustering and decision trees in understanding treatment outcomes, while combining extensive clinical and genetic data in a relatively homogenous cohort of pediatric patients with asthma, with a long-term clinical follow-up (2 years), which has not been done before.

## Population and Methods

### Population studied: The SCH (Srebrnjak Children`s Hospital) cohort

In this cohort there are 365 pediatric patients (355 children aged 2–17 years and 10 adolescents aged 18–22 years) with atopic and non-atopic, intermittent to severe persistent asthma [14], which were recruited in a prospective, non-interventional type of clinical study at the outpatient clinic at the Srebrnjak Children's Hospital (SCH). This cohort was also subject to our previous study [16]. Informed consent was obtained from the children's parents/legal guardians. The study protocol was approved by the local Ethics Committee (at SCH). Relevant clinical and other characteristics of the cohort (at baseline) are presented in Table 1.

**Table 1:** Clinical and other relevant characteristics (demographic, lung function, asthma features, comorbidity etc.) of the cohort (at baseline). SD- standard deviation, M- male, F- female, BMI- body mass index, AR- allergic rhinitis, AD- atopic dermatitis, GERD- gastroesophageal reflux disease, RI- reflux index, OSA- obstructive sleep apnoea, AHI- apnoea/hypopnea index, IgE- immunoglobulin E, WBC- white blood cells, hsCRP- high-sensitivity C-reactive protein. Percentile of BMI- underweight (≤ 5), normal (5–85), overweight (86–94), obese (≥ 95)

**Age (years)- (mean, SD)**	9.97 (3.97)	
	M- 9.68 (3.93)	F- 10.44 (4.01)
**Gender (M/F)- N (%)**	M 223 (61.10)	F 142 (38.90)
**Percentile of BMI- N (%)**	Underweight	11 (3.01)
	Normal	253 (69.32)
	Overweight	50 (13.70)
	Obese	51 (13.97)
**Duration of disease (years)- mean (SD)**	3.27 (2.83) *N* = 302	
**Comorbidities- N (%), mean (SD)**	AR	312 (85.48)
	AD ever	101 (27.67)
	AD currently	23 (6.33)
	GERD, RI score	101 (27.82), 9.10 (SD- 11.19)
	OSA, AHI	14 (3.84), 0.57 (SD- 2.84)
**Atopy (yes/no)- N (%)**	Yes	319 (87.40)
	No	46 (12.60)
**Total IgE (kIU/l)- mean (SD)**	614.54 (1145.62), *N* = 351	
**Eosinophil count (absolute)- median (SD)**	416.35 (341.27), *N* = 355	
**Neutrophil count (% of total WBC)- mean (SD)**	49.76 (12.86), *N* = 364	
**hsCRP (mg/l)- mean (SD)**	2.23 (9.30), *N* = 311	
**Lung FENO (ppb)- mean (SD)**	20.49 (20.07), *N* = 350	
**% of FEV**_**1**_ ** predicted- mean (SD)**	87 (17.14)	
**% of MEF**_**50**_ ** predicted- mean (SD)**	88 (23.11)	

At their first visit patients underwent physical examination, anthropometric measurements and a standard battery of diagnostic procedures and measurements to establish a diagnosis of asthma (lung function and allergy tests, as well as other tests and procedures- hematologic and biochemical blood tests, comorbidity testing etc.). The patients started treatment with inhaled corticosteroids, ICS (alone or in combination with LABA- long-acting beta-agonists) and/or LTRA (leukotriene receptor antagonists), according to GINA guidelines (Global Strategy for Asthma Management and Prevention, steps 1–5, according to presenting symptoms and assessed disease severity [14]). Treatment was prescribed by pediatric allergy or pulmonology specialists (study investigators) at SCH. Follow-up visits with lung function and airway inflammation testing as well as physical examination were made on average every 6 months over the period of 2 years (shown in Table 2). Additionally, treatment outcomes (responses) and the level of disease control (according to GINA guidelines) were assessed at each visit and if needed, treatment was adjusted according to the stepwise approach to asthma management [14]. The observational study is described in the supplementary file in detail.

**Table 2:** The features used in this study. The features are described into more detail in the supplementary file

baseline demographics	gender, age
subjective clinical data	at baseline (personal and family medical history- atopy status, allergic rhinitis (AR), atopic dermatitis (AD), food allergy and other comorbidities)
objective clinical data	at baseline and other follow-up appointments- symptom control, frequency and severity of exacerbations in the period since the last visit, lung function, airway inflammation (FENO) measurement and medication useat baseline- skin prick and total and specific IgE test results, hematologic and biochemical blood test results, comorbidity status ENT examination, pH probing with impedance for the reflux episodes monitoring for diagnostics of GERD/LPR, polysomnography for diagnostics of OSAS, height and weight for calculation of BMI percentiles
genetic data	genotypes for rs37973 (*GLCCI1*), rs9910408 (*TBX21*), rs242941 (*CRHR1*), rs1876828 (*CRHR1*), rs1042713 (*ADRB2*) and rs17576 (*MMP9)*

### Response variables

According to their response to treatment (at each visit, short-term- every 6 months and long-term- 12 and 18 months after treatment initiation), patients were divided into “good”, “moderate” and “poor” responders in accordance with the Minimal Clinically Important Difference (MCID) for lung function adjusted for children and data from other studies evaluating treatment response in asthma, taking into account changes in the level of disease control and changes in the level of airway inflammation- FENO values, presented in Table 3 [14, 17–21].

**Table 3:** Response variables assessed at each visit (compared to a previous one- 6, 12 and 18 months after baseline). Response to treatment is defined into more detail in the supplementary file (Table S3). ppb- parts per billion

	**FEV**_**1**_	**MEF**_**50**_	**FENO**	**Asthma control**
Good	Increase ≥ 10% predicted	Increase ≥ 15% predicted	Decrease < 20% for FENO values > 35 (50) ppb or increase < 10 ppb for FENO values < 35 (50) ppb	improvement in asthma control
Moderate	± 9% predicted	± 14% predicted	≤ 20% FENO ≤ 20% for FENO values over 35 (50) ppb or ± 10 ppb for FENO values < 35 (50) ppb	no changes in partial asthma control
Poor	Decrease ≤ (-10%) predicted	Decrease ≤ (-15%) predicted	Increase > 20% for FENO values > 35 (50) ppb or increase > 10 ppb for FENO values < 35 (50) ppb	deterioration in asthma control

### Machine learning and statistical methods

The data preprocessing is described in the supplementary file. Due to missing data 347 patients were included in the analysis. Hierarchical clustering analysis (HCA) on the response data was performed using the Ward’s method [5, 7, 8]. Clustering was performed on the patients` response data in each treatment phase from baseline to the 3rd control visit, represented as nominal data (1 = , 2 = , 3 = , corresponding to good, moderate and poor response to treatment, respectively). To determine the differences between the clusters we applied the Kruskal–Wallis test for continuous and the chi-square test for categorical variables [5]. Decision tree classification (DTC) [22] was used to reveal discriminatory phenotypic characteristics affecting response clustering based on non-linear relationships. Decision trees have proven useful for decision making [16, 23] often resembling human-like logic by binning patients according to their diagnostic features and are accepted by medical personnel [5]. The 4 clusters obtained from HCA on response outcomes were set as target classes (4 classes) for DTC. The features were all relevant data from baseline, as indicated in Table 2. The DTC algorithm provides feature importance, a non-linear technique for understanding machine learning decisions and prioritization of variables [16, 24–26] in our case important to differentiate among the clusters/classes.

## Results

We have identified 4 distinct outcome clusters from the dendrogram in Fig. 2 which are described in Table 4.

**Table 4:** Description of the obtained clusters from Fig. 1. The descriptions are extracted from the statistical analysis in Table 5

Cluster 1(*N* = 58)	patients with overall good response to treatment according to the level of asthma control, moderate response to treatment according to lung function parameters (relative changes in FEV_1_ and MEF_50_) and moderate or poor response to treatment according to changes in FENO
Cluster 2(*N* = 87)	patients with overall good response to treatment according to the level of asthma control, good or moderate levels of response to treatment according to changes in FENO, moderate response to treatment according to % change in FEV_1_ and poor or moderate levels of response to treatment according to changes in MEF_50_
Cluster 3(*N* = 138)	patients with overall good response to treatment according to all parameters analyzed (see Sect. 2.2.)
Cluster 4(*N* = 64)	patients with overall poor (moderate and poor) response to treatment according to changes in lung function, moderate levels of response according to FENO changes and long-term poor response to treatment according to the level of asthma control

The relevant features corresponding to outcome data and clinical, demographic and genetic data at baseline characterizing each response cluster/class (cluster statistics) are shown in Tables 5 and 6, respectively, while the main discriminants according to the DTC are presented by feature importance in % (see supplementary data, Table S5).

**Table 5:** Response to treatment (outcome)- related cluster statistics. Ward`s Euclidean method, χ2 test, p < 0.05. Abbreviations for respective responses to treatment are defined in Supplement Table 1

Feature / No of patients	Cluster 1 (*N* = 58)			Cluster 2 (*N* = 87)			Cluster 3 (*N* = 138)			Cluster 4 (*N* = 64)			*p*-value
	Good N (%)	Moderate N (%)	Poor N (%)	Good N (%)	Moderate N (%)	Poor N (%)	Good N (%)	Moderate N (%)	Poor N (%)	Good N (%)	Moderate N (%)	Poor N (%)	
Resp FEV_1_ baseline to 1^st^ control	17 (29.31)	27 (46.55)	14 (24.14)	8 (9.2)	45 (51.72)	34 (39.08)	75 (54.35)	49 (35.51)	14 (10.14)	19 (29.69)	24 (37.5)	21 (32.81)	< 0.001
Resp FENO baseline to 1^st^ control	1 (1.72)	57 (98.28)	0 (0)	79 (90.8)	7 (8.05)	1 (1.15)	108 (78.26)	14 (10.14)	16 (11.59)	58 (90.62)	4 (6.25)	3 (3.12)	< 0.001
Resp CTRL baseline to 1^st^ control	34 (58.62)	12 (20.69)	12 (20.69)	55 (63.22)	22 (25.29)	10 (11.49)	94 (68.12)	16 (11.59)	28 (20.29)	34 (53.12)	11 (17.19)	19 (29.69)	< 0.05
Resp MEF_50_ baseline to 1^st^ control	18 (31.03)	22 (37.93)	18 (31.03)	5 (5.75)	43 (49.43)	39 (44.83)	75 (54.35)	43 (31.16)	20 (14.49)	20 (31.25)	22 (34.38)	22 (34.38)	< 0.001
Resp FEV_1_ 1^st^ to 2^nd^ control	9 (15.52)	46 (79.31)	3 (5.17)	10 (11.49)	64 (73.56)	13 (14.94)	44 (31.88)	70 (50.72)	24 (17.39)	18 (28.12)	40 (62.5)	6 (9.38)	< 0.001
Resp FENO 1^st^ to 2^nd^ control	48 (82.76)	10 (17.24)	0 (0)	55 (63.22)	21 (24.14)	11 (12.64)	84 (60.87)	30 (21.74)	24 (17.39)	39 (60.94)	14 (21.88)	11 (17.19)	< 0.05
Resp CTRL 1^st^ to 2^nd^ control	52 (89.66)	2 (3.45)	4 (6.9)	59 (67.82)	11 (12.64)	17 (19.54)	118 (85.51)	4 (2.9)	16 (11.59)	46 (71.88)	6 (9.38)	12 (18.75)	< 0.01
Resp FEV_1_ 2^nd^ to 3^rd^ control	3 (5.17)	45 (77.59)	10 (17.24)	11 (12.64)	73 (83.91)	3 (3.45)	21 (15.22)	89 (64.49)	28 (20.29)	4 (6.25)	42 (65.62)	18 (28.12)	< 0.001
Resp FENO 2^nd^ to 3^rd^ control	16 (27.59)	26 (44.83)	16 (27.59)	29 (33.33)	48 (55.17)	10 (11.49)	54 (39.13)	75 (54.35)	9 (6.52)	23 (35.94)	29 (45.31)	12 (18.75)	< 0.01
Resp CTRL 2^nd^ to 3^rd^ control	53 (91.38)	3 (5.17)	2 (3.45)	82 (94.25)	5 (5.75)	0 (0)	128 (92.75)	6 (4.35)	4 (2.9)	2 (3.12)	9 (14.06)	53 (82.81)	< 0.001
Resp MEF_50_ 2^nd^ to 3^rd^ control	3 (5.17)	41 (70.69)	14 (24.14)	20 (22.99)	57 (65.52)	10 (11.49)	38 (27.54)	76 (55.07)	24 (17.39)	4 (6.25)	36 (56.25)	24 (37.5)	< 0.001
Resp FEV_1_ baseline to 2^nd^ control	22 (37.93)	23 (39.66)	13 (22.41)	6 (6.9)	42 (48.28)	39 (44.83)	79 (57.25)	54 (39.13)	5 (3.62)	19 (29.69)	30 (46.88)	15 (23.44)	< 0.001
Resp FENO baseline to 2^nd^ control	7 (12.07)	0 (0)	51 (87.93)	13 (14.94)	65 (74.71)	9 (10.34)	25 (18.12)	90 (65.22)	23 (16.67)	14 (21.88)	42 (65.62)	8 (12.5)	< 0.001
Resp CTRL baseline to 2^nd^ control	50 (86.21)	6 (10.34)	2 (3.45)	61 (70.11)	16 (18.39)	10 (11.49)	120 (86.96)	10 (7.25)	8 (5.8)	42 (65.62)	10 (15.62)	12 (18.75)	< 0.01
Resp MEF_50__diagn to 2^nd^ control	17 (29.31)	27 (46.55)	14 (24.14)	3 (3.45)	36 (41.38)	48 (55.17)	74 (53.62)	60 (43.48)	4 (2.9)	14 (21.88)	35 (54.69)	15 (23.44)	< 0.001
Resp FEV_1_ baseline to 3^rd^ control	19 (32.76)	20 (34.48)	19 (32.76)	8 (9.2)	49 (56.32)	30 (34.48)	71 (51.45)	62 (44.93)	5 (3.62)	8 (12.5)	39 (60.94)	17 (26.56)	< 0.001
Resp FENO baseline to 3^rd^ control	12 (20.69)	25 (43.1)	21 (36.21)	15 (17.24)	56 (64.37)	16 (18.39)	34 (24.64)	86 (62.32)	18 (13.04)	10 (15.62)	37 (57.81)	17 (26.56)	< 0.01
Resp CTRL baseline to 3^rd^ control	53 (91.38)	5 (8.62)	0 (0)	81 (93.1)	6 (6.9)	0 (0)	135 (97.83)	3 (2.17)	0 (0)	4 (6.25)	20 (31.25)	40 (62.5)	< 0.001
Resp MEF_50_ baseline to 3^rd^ control	11 (18.97)	30 (51.72)	17 (29.31)	5 (5.75)	37 (42.53)	45 (51.72)	71 (51.45)	61 (44.2)	6 (4.35)	4 (6.25)	36 (56.25)	24 (37.5)	< 0.001

**Table 6:** Cluster statistics related to relevant clinical, demographic and genetic data, including treatment use at baseline. Ward`s Euclidean method, Kruskal- Wallis and χ2 test, *p* < 0.05. Strong sensitization defined as sIgE to respective allergen of > 17.51 kU/L (classes 4–6)

Feature	Cluster 1	Cluster 2	Cluster 3	Cluster 4	*p* value
No of patients	*N* = 58	*N* = 87	*N* = 138	*N* = 64	
Strong sensitization to house dust mite (sIgE d1)—No	18 (31.03%)	40 (45.98%)	73 (52.90%)	31 (48.44%)	< 0.05
Strong sensitization to house dust mite (sIgE d1)—Yes	40 (68.97%)	47 (54.02%)	65 (47.10%)	33 (51.56%)	< 0.05
	Mean (STD)	Mean (STD)	Mean (STD)	Mean (STD)	
% FEV1 predicted at baseline	0.89 (0.20)	0.96 (0.13)	0.82 (0.17)	0.88 (0.15)	< 0.001
% MEF50 predicted at baseline	0.92 (0.22)	1.03 (0.19)	0.79 (0.22)	0.84 (0.24)	< 0.001
FENO at baseline (ppb)	21.5 (23.61)	17.23 (16.15)	23.07 (23.14)	18.59 (13.38)	
Age (yrs)	11.79 (3.39)	10.16 (3.82)	9.98 (3.84)	9.61 (3.64)	< 0.01
Height (cm)	153.05 (17.78)	143.9 (19.11)	143.86 (21.12)	142.23 (21.24)	< 0.05
Disease duration (yrs)	5.72 (3.43)	5.08 (3.84)	4.12 (3.8)	3.45 (3.11)	< 0.001
Total IgE (kIU/L)	686.34 (744.36)	421.95 (578.5)	653.9 (1164.19)	484.79 (516.04)	
Eosinophil absolute count (Dunger)	419.99 (284.96)	418.71 (347.17)	380.16 (305.29)	377.22 (349.65)	
Neutrophil blood count (%)	49.72 (11.26)	50.79 (13.18)	49.57 (12.82)	51.99 (12.96)	
hsCRP (mg/L)	2.58 (6.31)	4.02 (14.45)	3.11 (7.52)	2.57 (3.72)	
Platelets (× 109/L)	291.09 (86.54)	278.68 (108.14)	283.72 (105.47)	257.7 (105.31)	
ICS medium and high doses use from 2nd to 3rd visit, N (%)	30 (51.72%)	32 (36.78%)	51 (36.96%)	38 (59.38%)	< 0.05
SABA use from 2nd to 3rd visit, N (%)	7 (12.07%)	6 (6.90%)	4 (2.90%)	27 (42.19%)	< 0.001
BMI percentile 0–5, N(%)	0 (0)	3 (3.45)	4 (2.9)	3 (4.69)	
BMI percentile 5–85, N(%)	47 (81.03)	53 (60.92)	94 (68.11)	46 (71.88)	
BMI percentile > 85, N(%)	11 (18.87)	31 (35.63)	40 (28.99)	15 (23.43)	
Gene_rs37973—GG	13 (22.41%)	13 (14.94%)	15 (10.87%)	12 (18.75%)	< 0.05
Gene_rs37973—GA	18 (31.03%)	48 (55.17%)	70 (50.72%)	34 (53.12%)	< 0.05
Gene_rs37973—AA	27 (46.55%)	26 (29.89%)	53 (38.41%)	18 (28.12%)	< 0.05

The main phenotype variable discriminatory for the response clusters according to DTC was MEF_50_ predicted at baseline, followed by the use of reliever medication (SABA) which is a parameter incorporated in asthma control assessment, use of combination treatment (ICS + LABA) which also indicates poorer disease control; hsCRP, FENO at baseline, neutrophil blood count which reflect the type and level of inflammation, and total IgE which corresponds to the atopy status and sensitization levels (see Fig. 2 and Table 6), although these variables were not significantly different between clusters in the cluster statistics.

## Discussion

Our results indicate that clusters 1–3 have overall good long-term treatment outcomes assessed by changes in asthma control. Cluster 1 had moderate levels of response to treatment according to lung function parameters (both FEV_1_ and MEF_50_), which may be explained by the fact that these patients didn`t have significantly impaired lung function at baseline. These patients also had relatively poor FENO- related response to treatment, which may be a consequence of sensitization to HDM, as the majority of these patients had strong sensitization to HDM (sIgE > 17.51 kU/L), see Tables 5 and 6. A study involving a pediatric cohort in Korea has demonstrated that the levels of sIgE to HDM correlate with increases in FENO [27]. Moreover, sensitization to HDM has been associated with poorer disease outcomes in children. [28] Also, these patients were older (mean age ca. 12 years) and had later onset of the disease (ca. 6 years of age), which may also contribute to poorer response to treatment [3–7]. Cluster 1 also had the highest eosinophil count and the highest serum total IgE levels (Fig. 2 and Table 6), which may indicate a higher level of Th2 inflammation.

Cluster 2 was similar to cluster 1 in terms of response to treatment according to disease control and FEV_1_ parameters, but they had good or moderate levels of response to treatment according to FENO changes, probably due to the fact that this cluster was not significantly associated with sensitization to HDM. These children had relatively earlier age of onset of disease (ca. 5 years of age). Additionally, cluster 2 patients had poor MEF_50_- related response, although their baseline MEF_50_ measurements were not impaired (Tables 5 and 6). This suggests that lung function in the distal airways deteriorates with time in these patients despite regular medication use which contributes to the importance of the small airways in children with asthma [29]. Additionally, there is evidence that obstruction in the small airways may be involved in the pathophysiology and resistance to treatment with ICS in children, especially those with increased BMI [30] and that the impairment of the small airways disease may be present despite rare and mild asthma symptoms and normal spirometry in children [31].

**Fig. 1:**
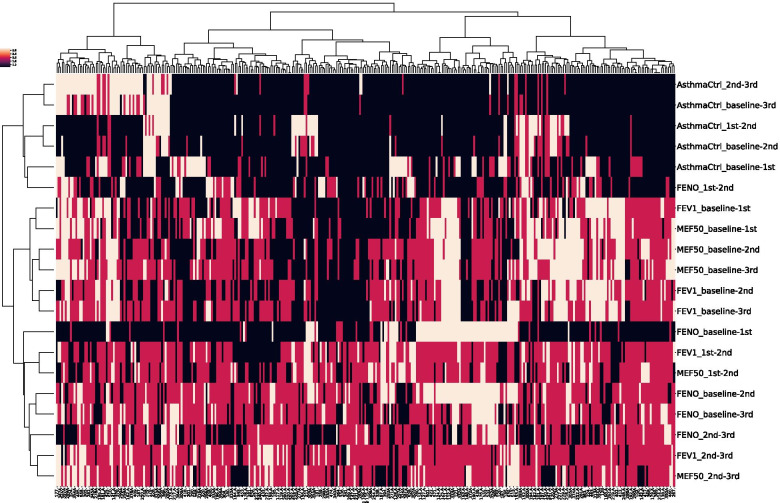
Hierarchical cluster analysis (HCA) of response to treatment with common classes of asthma treatment. *N* = 347, 12 features used: response to treatment according to changes in MEF_50_, FENO, FEV_1_ and level of disease control between each respective visit (baseline to 3rd control visit)

**Fig. 2:**
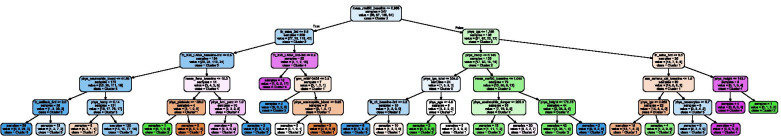
Main discriminants (relevant features) characterizing each outcome (response) clusters/class corresponding to clinical, demographic and genetic data at baseline, according to the decision tree algorithm. Ward`s method, p < 0.05, Gini < 0.2. The short/long names for respective variables are defined in the supplementary data (Table S4)

Cluster 2 had the highest levels of serum hsCRP (Fig. 2 and Table 6), which indicates that these patients may have higher levels of systemic inflammation and hence, poorer disease and treatment outcomes. [32] Moreover, cluster 2 patients had a higher proportion of overweight and obese patients compared to other clusters (Table 6, Fig. 2), which is in concordance with other findings indicating that obesity in asthma is associated with poorer disease outcomes and non-responsiveness to treatment with ICS. [33, 34] These patients also had higher levels of eosinophilic inflammation (eosinophil count) than clusters 3 and 4 but also higher neutrophil count than clusters 1 and 3 (Table 6, Figs. 1 and 2), supporting recent findings that obesity in mice is associated with a mixed granulocytic inflammation and may contribute to a refractory therapeutic response as well as exacerbation of disease severity [35].

Cluster 1 was also different from cluster 2 in exhibiting a dominant genotype (AA) and allelic (A allele) effect for the rs37973 polymorphism in the *GLCCI1* gene, previously associated with positive treatment outcomes in patients using ICS (Table 6). Also, clusters 1 and 3 differ from 2 and 4 in rs37973 distribution.

Cluster 3 were somewhat younger than patients in clusters 1 and 2 (mean age just under 10 years) but still had a relatively later onset of disease (ca. 6 years of age). These patients had the lowest FEV_1_ and MEF_50_ at baseline measured (Tables 5 and 6), which indicates that they had the highest improvement in lung function in response to treatment. These patients also had a higher frequency of the A allele for rs37973, which may contribute to better responsiveness to ICS (Table 6) [36]. Hence, clusters 1 and 3 have very similar frequencies of alleles and genotypes, while clusters 2 and 4 have very similar frequencies of alleles and genotypes. Allele A is highly overrepresented in cluster 1 and 3 in comparison to 2 and 4. Cluster 3 was also characterized by higher serum total IgE levels (Table 6, Fig. 2), but not with significantly higher eosinophil or neutrophil count, which may indicate lower levels of airway inflammation in these patients contributing to positive treatment outcomes. Additionally, these patients had the highest levels of FENO at baseline (see Table 6, Figs. 1 and 2), which might explain their better responsiveness to treatment with ICS [37].

Cluster 4 was the only one characterized by poor long-term control-related response. Additionally, these patients had poor treatment outcomes according to lung function parameters, in spite of the highest reliever medication use and highest rate of medium and high ICS doses use of all clusters (Table 5). These patients were the youngest (mean age 9.6 years) but also had later onset of disease (ca. 6 years of age). They had somewhat lower FEV_1_ and MEF_50_ measurements at baseline, but still within acceptable physiologic range (Table 6), indicating lung function impairment with time. Cluster 4 patients had the highest neutrophil count (Fig. 2, Table 6), which has been associated with more severe asthma outcomes and, moreover, with non-responsiveness to corticosteroids [38]. Additionally, cluster 4 had lower platelet counts compared to other clusters (Table 6). Lower platelet count due to their contribution to allergic inflammation might be more prominent in children [39]. Platelets may also be involved in more extensive airway remodeling, as well as in the development of steroid-refractory asthma, since ICS do not affect platelet function [40].

Although a number of clustering studies have performed unbiased statistically based analyses on large cohorts of patients involving a wide range of clinical variables, they have been limited in the terms of clinical characteristics they have used to identify different phenotypes and still do not provide much insight into the underlying disease mechanisms [2]. Additionally, different methods employed in these studies have been shown to yield different results in cluster assignments, especially in different populations [41, 42]. To the best of our knowledge, this is the first study focusing on treatment outcome patterns and response to treatment in children and the pathophysiological mechanisms underlying such outcomes. To date, only one study has focused on long-term treatment outcomes in 3 independent cohorts (including pediatric patients) [15]. A limitation of the present study is that these findings may very well be population-specific. The study population was very homogeneous (mostly milder disease forms, mostly atopic, ethnically homogeneous), which was an advantage in identifying genetic traits associated with treatment response patterns, but a disadvantage in identifying clear disease phenotypes. Also, since some children with asthma are known to “switch” phenotypes during the course of their disease, it is not certain whether these results reflect a current state (transient phenotype) or a stable sum of clinical manifestations and disease traits underlying specific (long-term) treatment outcome patterns [43]. Additionally, the treatment outcome assessment period may have been too short to reflect any biologically significant effects, especially on complex traits such as lung function changes in response to treatment. On the other hand, the latest control-based GINA guidelines suggest treatment response review every 3–6 months and longer-term assessment (such as the one in this study) will minimize possible random effects when focusing on shorter periods of treatment use. Although the total number of variables used in this study was large (*N* = 280), surely not all clinically significant traits were encompassed and additionally, we could only infer on certain pathophysiologic mechanisms indirectly. We did not use direct biomarkers of airway inflammation, such as induced sputum or bronchoalveolar lavage (BAL), but in pediatric cohorts minimally invasive procedures are an absolute prerequisite. This is why we used surrogate biomarkers- blood eosinophil and neutrophil count as well as FENO level. Recent findings suggest that blood eosinophil count is a simple and valid biomarker in the management of asthma, reliably predicting future risk of exacerbations and treatment response [44]. Additionally, the sample size in certain subgroups (clusters) might be small, preventing more detailed phenotype characterization.

## Conclusion

**Fig. 3:**
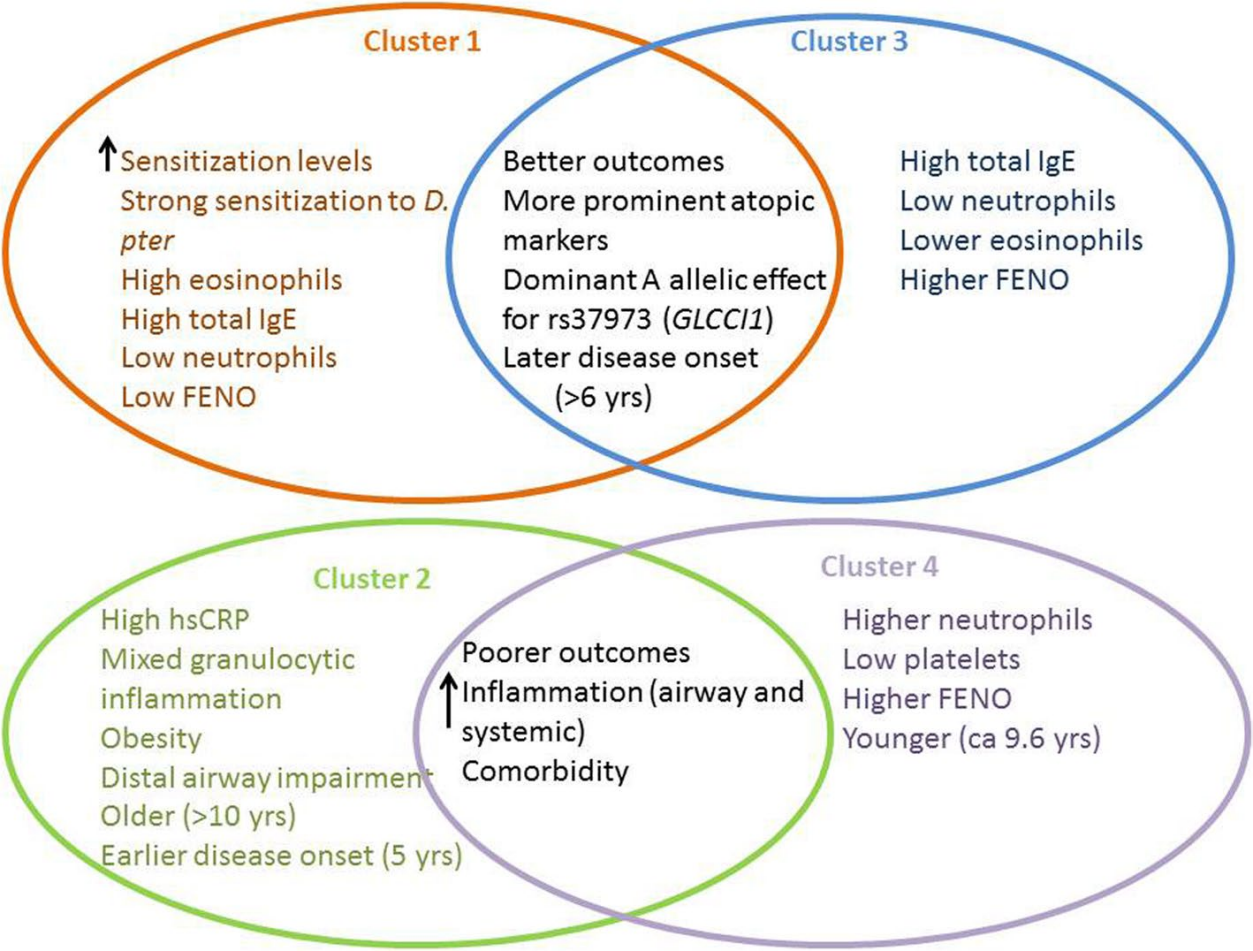
A schematic representation of the main characteristics of the 4 clusters identified in this study. Clusters 1 and 3 seem to have a more positive pattern of treatment outcomes and were characterized by more prominent atopic markers and a predominant allelic (A) effect for rs37973, a polymorphism in the GLCCI1 gene, and with a relatively later onset of disease. Clusters 2 and 4 had poorer treatment success patterns and were characterized by higher levels of airway and systemic inflammation and comorbidities, but varied in the type of inflammation (predominantly neutrophilic for cluster 4 and mixed-type for cluster 2) and platelet count (lowest for cluster 4). Cluster 2 was the only one with relatively earlier onset of asthma (5 years of age)

We have identified 4 distinct response clusters varying in treatment outcomes according to lung function, airway inflammation and disease control parameters and duration of treatment, briefly presented in Fig. 3.

The results of this study underpin the issues in asthma treatment and management due to the overgeneralized approach to the disease, not taking into account specific disease phenotypes in children. The cohort will be followed up additionally, both for cluster (phenotype) stability and transitions as well as to compare (confirm) these findings in other age groups and populations. Further characterization of specific disease phenotypes is essential, involving larger numbers of patients, multi-centric, longitudinal and prospective studies and even more clinically relevant parameters. Additionally, it is of high importance to distinguish between meaningful asthma subtypes at a population and individual patient level, and to identify specific mechanisms and novel endotypes involved in the disease presentation in order to develop personalized treatment as well as prevention strategies. This will aid in developing complex prediction models which will stratify patients according to their specific disease traits and risk for treatment failure, potentially establishing novel and better therapeutic options and enabling full quality of life for patients with asthma.

## Supplementary Information

**Additional file 1.**

**Additional file 2.**

## Data Availability

Not applicable.

## Abbreviations

AD: Atopic dermatitis
ADRB2: Beta-2 adrenergic receptor
AR: Allergic rhinitis
BMI: Body mass index
CRHR1: Corticotropin Releasing Hormone Receptor 1
ENT: Ear/nose/throat
FENO: Fraction of exhaled nitric oxide
FEV1: Forced expiratory volume in one second
FVC: Forced vital capacity
GERD: Gastroesophageal reflux disease
GINA: Global Initiative for Asthma
GLCCI1: Glucocorticoid Induced 1
HDM: House dust mite
hsCRP: High-sensitivity C-reactive protein
ICS: Inhaled corticosteroids
IgE: Immunoglobulin E
LABA: Long-acting beta agonists
LOAC: Level of asthma control
LPR: Laryngopharyngeal reflux
LTRA: Leukotriene receptor antagonists
MCID: Minimal clinically important difference
MEF50: Maximal Expiratory Flow at 50%
MMP9: Matrix metalloproteinase-9
NO: Nitric oxide
OSAS: Obstructive sleep apnoea syndrome
ppb: Parts per billion
SNP: Single nucleotide polymorphism
SPT: Skin prick test
TBX21: T-box 21
Th2: T-helper 2
