# Supplementary material for: Treatment outcome clustering patterns correspond to discrete asthma phenotypes in children
Source: Asthma Res Pract. 2021 Aug 3;7:11. doi: 10.1186/s40733-021-00077-x (PMC8330019; doi:10.1186/s40733-021-00077-x)
Supplement: Supplementary file 1 — Additional file 1. [file 40733_2021_77_MOESM1_ESM.docx]

**Supporting information**

**Supporting materials for Banić et al, 2021****.**

Contents

[Observational study description 1](#_Toc64274760)

[Inclusion 2](#_Toc64274761)

[Exclusion criteria 3](#_Toc64274762)

[Follow up 4](#_Toc64274763)

[Assessments 4](#_Toc64274764)

[Potential bias 5](#_Toc64274765)

[Diagnostic data description 5](#_Toc64274766)

[Assessment and biochemistry 5](#_Toc64274767)

[Genetic analysis 6](#_Toc64274768)

[Definition of response to treatment variables 8](#_Toc64274769)

[Data preprocessing 10](#_Toc64274770)

[Table 11](#_Toc64274771)

[Supplement literature 23](#_Toc64274772)

# Observational study description

The controlled and observational (non-interventional) 24-months study with prospective cohort designs and longitudinal data collection (data collected on average every 6 months) was performed in pediatric patients with physician established diagnosis of asthma. The routine standardized follow-up assessments of all patients included - First patient (or study subject), First Visit (FPFV): 1 month after the beginning of the study - Last Patient (or study subject), First Visit: 1-6 months - Last Patient (or study subject), Last Visit: 24-30 months - End of Study Including follow up and data analysis); 30 months. The study size was estimated at 400, with a dropout rate after the 24-month follow-up period being ca. 10% (N=365).

## Inclusion

The study includes patients who signed informed consent, i.e. agreed to participate in a clinical study and meet the criteria for inclusion. Criteria for inclusion were assessed by a specialist physician (pediatric allergy or pulmonology specialist). The children were enrolled in the study after their legal guardians have read, agreed to, and signed the informed consent form. All children older than 12 as well as adolescents were enrolled in the study after signing the inform consent, as well. The subjects were assessed for inclusion and exclusion criteria on first study visit after signing the informed consent. Patients involved in the study used their regular therapy (according to guidelines) i.e. conduct treatment and all other non-therapeutic measures as if they were not involved in the study (real life study). Any deterioration or emergency interventions were evaluated and recorded as additional visits. The new therapies were not applied to study patients during the observational period and the patients continued with their standard therapy (according to GINA guideline) as if they were not included in the study. The patients with a clinical diagnosis of asthma (according to ERS/ATS guidelines) for at least one year [1], being on a stable dose of anti-inflammatory treatment for at least one month with partially controlled or uncontrolled asthma according to GINA guidelines were recruited from the SCH outpatient clinic during their regular visit to the clinic (real life study). Additional inclusion criteria were clinically significant allergy to indoor and outdoor allergens, with positive skin prick test (SPT) and specific IgE levels (>0.7kUA/L). SPT was performed to a standard palette of inhaled allergens containing: birch, grass pollen, mugwort, Ambrosia elatior, Dermatophagoides pteronyssinus (Der p), Dermatophagoides Farinae (Der f), dog, cat, hazel, Cladosporium, Alternaria negative control and histamine (Allergopharma, Reinbek, Germany).

Additionally, if indicated, patients underwent SPT to food allergens: eggs, cow's milk, wheat flour, soy, peanut, fish, nuts and sesame, as well as other food allergens (if indicated). The interpretation of skin prick test results (based on the guidelines of the European Academy of Allergy and Clinical Immunology were made after approximately 15–20 min in relation to positive (the histamine dihydrochloride at a concentration of 1.0 mg/ml) and negative control (saline). The diameter of urtica or wheal diameter of at least 3 mm was considered a positive result, according to the EAACI recommendation. [2] Allergen solutions were tested on the volar surface of the forearm using 1mm prick lancets. The SPT was not repeated if the respondent had a test finding that was not older than one year.

## Exclusion criteria

Exclusion criteria for adolescents were: acute respiratory infection, use of systemic corticosteroids, recent asthma-related visit to emergency department (in the past three weeks) and coexistence of other serious chronic illness. Exclusion criteria for school children were: known inborn or perinatal pulmonary disease; pulmonary malformation; oxygen therapy after birth with a duration of more than 24 h; ventilator support or mechanical ventilation after birth; diagnosis of cystic fibrosis; primary ciliary dyskinesia; heart failure diagnosed after birth affecting pulmonary circulation; major respiratory diseases such as e.g. interstitial lung disease. Moreover, children were excluded from study visits and biomaterial collection in the case of fever of at least 38.5 °C during the last two weeks prior to the planned visit.

## Follow up

All patients were regularly followed up on average every 6 months and regularly controlled even more often if they had a history of exacerbations. Lung function testing, FENO and asthma control (including C-ACT score, if available- N= 158) were recorded and assessed at every visit.

## Assessments

The childhood asthma control test (C-ACT) is a brief, patient-administered asthma assessment tool that helps healthcare professionals quickly and effectively assess asthma control in patients. It was clinically validated by assessment and spirometry and is able to detect changes in asthma control over time to help determine whether or not a patient’s current treatment plan is working. It consists of seven questions, with a minimum score of 0 and maximum of 27. Higher score represents better level of control. The cutoff value of 19 points represents inadequate asthma control. Lung function (school aged children) by standard spirometry was performed according to the ATS/ERS spirometry standards. We measured forced vital capacity (FVC), forced expiratory volume at the first second (FEV1), FEV1/FVC ratio and mid forced expiratory flow (FEF25-75) and express values as absolute ones and as percentage of the predicted values according to Quanjer. FENO (for school age children and adults). Fraction of exhaled nitric oxide (FENO) was measured using a chemiluminescence analyzer (NIOX or MEDISOFT analyzer) during a single-breath exhalation according to the ERS/ATS recommendations at a flow rate of 50 mL/sec.

## Potential bias

Potential bias included overrepresentation of one gender (male) since childhood asthma is more common in boys. During the recruitment procedure, children were included in the study regardless of their gender, if they met the inclusion criteria. Additionally, to avoid under- or over-estimation of the disease diagnosis and state (disease severity and control), the recruitment was performed by experienced clinicians (allergy specialist with 15+ years of experience in the field). Although children of all age groups (3-18) were recruited to the study if they met the inclusion criteria, a diagnosis of asthma was difficult to establish in young children (wheeze, transient wheeze and other differential diagnoses), a potential bias is underrepresentation of younger children (up to 5 years of age).

# Diagnostic data description

## Assessment and biochemistry

At their first visit patients underwent physical examination, anthropometric measurements (height and weight for calculation of BMI and BMI percentiles), along with a standard battery of diagnostics procedures and measurements to establish a diagnosis of asthma. These included skin prick tests for common allergens (list in supplementary data), lung function tests (including airway challenge tests, if applicable) and blood sampling for routine laboratory diagnostics (hematology, biochemistry and allergy assays- total and allergen- specific IgE). In order to assess the level and type of both systemic and local inflammation, certain inflammatory biomarkers were measured at baseline in all participants, including fractional exhaled nitric oxide (FENO), high-sensitive C-reactive protein (hsCRP) as well as certain inflammatory cell counts, such as eosinophils and neutrophils. [3,4] In order to identify additional conditions that might affect and aggravate the underlying disease (and asthma control), the participants were tested for common asthma comorbidities, such as gastroesophageal reflux disease (GERD), obstructive sleep apnea syndrome (OSAS) as well as allergic rhinitis and atopic dermatitis, including anamnesis data taken from the child`s parents. [5,6] Peripheral whole blood samples were collected by venipuncture into EDTA coated vacutainers (for hematology analyses) and into vacutainers with clot activator and gel for serum separation (for biochemistry and certain allergy assays). During this study a total of 10,5 ml peripheral blood samples per participant maximum was collected at baseline visit (recruitment point). The remainder of blood samples (in EDTA coated vacutainers) and sera left over after diagnostic tests was stored at -20°C for subsequent analyses, including genotyping.

## Genetic analysis

All genetic loci analysed in this study were chosen due to their previous association with treatment outcomes in patients with asthma (and childhood asthma, where applicable) in an effort of replicating these associations.

Genomic DNA was extracted from EDTA-containing peripheral whole blood samples, previously stored at -20°C, using QIAamp DNA Blood Mini Kit (Qiagen GmbH, Germany) according to the manufacturer’s instructions, by an automated (Qiacube, Qiagen GmbH, Germany) or manual spin protocol. The quality and concentration of each DNA isolate was checked using a NanoDrop^™^ 2000 spectrophotometer (ThermoFisher Scientific Inc., USA). The genotypes of the SNPs analyzed were determined using a 5`-nuclease allelic discrimination assay in a 96-well format and Taqman technology. Primers and probes were purchased from Applied Biosystems (Life Technologies, USA) for SNP genotyping assays rs37973 in *GLCCI1*, rs9910408 in *TBX21* and rs242941 and rs1876828 in *CRHR1*, as well as rs1042713 in *ADRB2*. Allelic discrimination assays were performed in 5 μL reaction volumes, using approximately 5 ng of DNA as a template, 2x TaqMan Fast Advanced Master Mix, and predesigned SNP genotyping assays provided by Applied Biosystems for rs37973, rs9910408, rs242941 and rs1876828. Temperature conditions for qPCR were set at 50°C for 2 minutes and 95°C for 20 seconds, followed by 40 cycles at 95°C for 3 seconds and at 60°C for 30 seconds. For rs17576 SNP (*MMP9*) genotyping a primer and probe set were designed using a free online software qPCR primer & probe design tool and design service (Eurofins Genomics, Germany). The forward and reverse primer as well as probes sequences are presented in Table 1.

Table S1. Primers (forward and reverse) and probes (allele 1 and allele 2) design for rs17576 genotyping assay (*MMP9* Gln279Arg, A/G transition detection). FWD- forward primer, REV- reverse primer, Allele 1 PR- probe for SNP allele 1, Allele 2 PR- probe for SNP allele 2, bp- base pairs (length of sequence in base paris), reporter- fluorescent dye, quencher- fluorescent dye quencher pair. For probe sequences nucleotides highlighted in red denote the ambiguity position (transition).

| **Oligonucleotide type** | **Length (bp)** | **Sequence** | **Reporter/quencher** |
| --- | --- | --- | --- |
| MMP9 Gln279Arg FWD primer | 19 | TCCCCCTTTCCCACATCCT |  |
| MMP9 Gln279Arg REV primer | 21 | CAGGGTTTCCCATCAGCATTG |  |
| MMP9 Gln279Arg Allele 1 PR | 17 | CTCTACACCC**A**GGACGG | VIC-BHQ1 |
| MMP9 Gln279Arg Allele 2 PR | 17 | TCTACACCC**G**GGACGG | FAM-BHQ1 |

Allelic discrimination assays for rs1042713 and rs17576 was performed in 12.5 μl reaction volume, using approximately 5 ng of DNA template (2x Brilliant III Ultra-Fast QPCR Master Mix (Agilent Technologies, USA), 100 mM of primer set (forward and reverse) and 10 mM of probes, with qPCR conditions as follows: 50°C for 2 minutes and 95°C for 10 minutes, followed by 40 cycles at 95°C for 15 seconds and at 60°C for 1 minute.

Genotyping of the amplified PCR products was determined by differences in VIC and FAM fluorescent levels, using the ABI Prism 7500 Fast Real-Time PCR system (system instrument equipped with SDS v2.0.5 software, Applied Biosystems, ThermoFisher Scientific Inc., USA) for rs37973, rs9910408, rs242941 and rs1876828 and using the Agilent AriaMX Real-Time PCR system (system instrument equipped with AriaMx software v1.0, Agilent Technologies, USA) for rs1042713 and rs17576.

### Genotyping results

Genotype distribution (frequency) for each genetic polymorphism is shown in Figure s1. All participants were successfully genotyped for rs37973, rs9910408, rs242941. For rs1042713 and rs17576 genotype data was missing for 1 and 19 participants, respectively, due to insufficient DNA extract material or degraded DNA samples in subsequent/ repeated analysis. Genotyping was performed at the Srebrnjak Children`s Hospital in Zagreb, Croatia in 2 batches on 2 different platforms.

Figure S1. Genotype frequency (%) for respective genetic polymorphisms: rs37973, rs9910408, rs242941, rs1876828, rs1042713 and rs17576. For rs37973, rs9910408, rs242941 and rs1876828 N=365, for rs1042713 N=364 and for rs17576 N=346.

Consistency with the Hardy- Weinberg equilibrium (HWE) for each genetic polymorphism, along with global and population-specific minor allele frequency (MAF) is presented in Table 14.

Table S2. HWE consistency for genotype frequencies for rs37973, rs9910408, rs242941, rs1876828, rs1042713 and rs17576. χ2- chi-squared value, p< 0.05 consistent with HWE. Global and population specific (Central European) MAF according to NCBI dbSNP (https://www.ncbi.nlm.nih.gov/SNP/). HWE- Hardy-Weinberg equilibrium, MAF- minor allele frequency, CEU- Central European, reference population. Calculated using Michael H. Court's (2005-2008) online calculator.

| **Genotype** | **rs37973** | | **rs9910408** | | **rs242941** | | **rs1876828** | | **rs1042713** | | **rs17576** | |
| --- | --- | --- | --- | --- | --- | --- | --- | --- | --- | --- | --- | --- |
|  | **Observed** | **Expected** | **Observed** | **Expected** | **Observed** | **Expected** | **Observed** | **Expected** | **Observed** | **Expected** | **Observed** | **Expected** |
| Homozygote reference | 130 | 130.2 | 103 | 106.9 | 150 | 155.2 | 270 | 270.1 | 113 | 88.0 | 50 | 83.0 |
| Heterozygote | 176 | 175.6 | 189 | 181.3 | 176 | 165.6 | 88 | 87.7 | 132 | 182.0 | 239 | 172.9 |
| Homozygote variant | 59 | 59.2 | 73 | 76.9 | 39 | 44.2 | 7 | 7.1 | 119 | 94.0 | 57 | 90.0 |
| Variant allele frequency | 0.40 | | 0.46 | | 0.35 | | 0.14 | | 0.51 | | 0.51 | |
| χ^2^ | 0.002 | | 0.664 | | 1.433 | | 0.003 | | 27.433 | | 50.508 | |
| p (1 degree of freedom) | 0.965 | | 0.415 | | 0.231 | | 0.956 | | 0.000 | | 0.000 | |
| Global MAF | 0.396 | | 0.384 | | 0.323 | | 0.086 | | 0.476 | | 0.456 | |
| CEU MAF | 0.442 | | 0.456 | | 0.282 | | 0.240 | | 0.358 | | 0.381 | |

## Definition of response to treatment variables

Table S3. Detailed explanation of how each response to treatment variable was defined.

| Lung function | **Poor response** to treatment according to changes in FEV_1_ was defined as a decrease in FEV_1_ predicted (for children of certain age, gender and posture) by 10% or more between clinical visits/follow-ups (≤10%). **Moderate response** to treatment according to changes in FEV_1_ was defined as a relative change in FEV_1_ predicted by ±9% and **good response** to treatment was defined as an increase in FEV_1_ predicted by 10% or more (≥10%) between clinical assessments/follow-ups.  **Poor response** to treatment according to changes in MEF_50_ was defined as a decrease in MEF_50_ predicted by 15% or more (≤15%); **moderate response** was defined as a relative change in MEF_50_ predicted by ±14% and **good response** to treatment was defined as an increase in MEF_50_ predicted by 15% or more (≥15%) between clinical visits/follow-ups. |
| --- | --- |
| Airway inflammation | We used cut-off points rather than reference values when interpreting FENO levels. **Poor response** to treatment was defined as an increase in FENO greater than 20% for values over 35 (50 for patients older than 18 years) ppb or more than 10 ppb for values lower than 35 (50) ppb between clinical visits. A reduction of at least 20% in FENO for values over 35 (50) ppb or more than 10 ppb for values lower than 35 (50) ppb was defined as the cut-off point to indicate a **significant (good) response** to anti-inflammatory treatment. **Moderate response** to treatment was defined as changes in FENO values ranging from a reduction of ≤20% and increase ≤20% for FENO values over 35 (50) ppb and ±10 ppb for values lower than 35 (50) ppb from one visit to the next. |
| Asthma control | The level of asthma control between clinical visits was assessed according to GINA guidelines: symptom occurrence (including nocturnal symptoms), need for reliever medications, number and severity of asthma exacerbations, lung function and Asthma Control Test (ACT), where applicable. The level of control was defined as either controlled, partly controlled or uncontrolled, whereas **poor response** to treatment was defined as deterioration in asthma control between visits, **good response** was defined as improvement in asthma control and **moderate response** was defined as no changes in partial asthma control between clinical visits, with the exception of the patient having uncontrolled asthma from visit to visit, which was considered a poor response or good response to treatment, when the patient had controlled asthma between visits. |

# Data preprocessing

All operations on data were performed either in R (<https://www.r-project.org/>) and Python (<https://www.python.org/>). Preprocessing, explorative analysis, feature selection and predictive models were done with the Python libraries pandas [7] , numpy [8] and scikit-learn [9]. Plotting was done using the libraries seaborn [10] and matplotlib [11]. At the 4th control visit, a significant proportion of the dataset had missing values due to the fact that not all patients were followed up to the 4th control visit (ca. 2 years after recruitment) or dropped out of the study. Some features had missing values due to patients` lack of cooperation (e.g. patient was too young to cooperate for lung function or FENO measurement) or there was insufficient blood/serum sample to perform certain biochemical tests. Rare which had missing values up to the 3rd control visit were imputed by their respective median for continuous features or mode for discrete features. The data was divided into treatment periods, i.e. baseline, first, second and third control visit. Features containing string notations (e.g. drug prescribed or genetic data) where numerically encoded and subsequently one-hot encoded. Features describing allergic sensitization were converted to binary or ordinal features, and the binary values were summed into in 4 clinically relevant categories: seasonal allergens (i.e. grass, weed and tree pollen), perennial allergens (house dust mite and molds), insect venom (bee, wasp or hornet) and food allergens. Both SPT results and specific IgE results were taken into account for each respective sensitization category. Additionally, certain sensitization features were assigned separate variables, due to their possible clinical relevance- strong sensitization to house dust mite- HDM (*D. pteronyssinus, D. farinae*), cat dander and ragweed (*Ambrosia*). These sensitizations have previously been associated with disease severity and more severe outcomes. [12–14] Strong sensitization was defined as sIgE class R4-R6 to the respective allergen (sIgE to the respective allergen > 17.50 kU/L). Atopic dermatitis was one-hot encoded, where values from -1 to .5 were assigned the value 0 and values above .5 were assigned a value of 1. Patients with missing response data (18 patients) were removed from the analysis. Feature selection for clustering was conducted through filtering based on the variance threshold, which was set to 5%.

# Table

Table S4. Short and long names for the variables included in the study

| alrg_atopy | Atopy status |
| --- | --- |
| alrg_d1_perennial_ige | sIge to Dermatophagoides pteronyssinus (d1) |
| alrg_d2_perrenial_ige | sIge to Dermatophagoides farinae (d2) |
| alrg_e1_perennial_ige | sIgE to cat dander |
| alrg_food_ige | sIgE to any food allergen |
| alrg_food_spt | SPT results to food allergens |
| alrg_perennial_ige | sIgE to any perennial inhaled allergen |
| alrg_perennial_spt | SPT results to any perennial inhaled allergen |
| alrg_season_ige | sIgE to any seasonal inhaled allergen |
| alrg_season_spt | SPT results to any seasoonal inhaled allergen |
| alrg_venom_ige | sIgE to Hymenoptera venom |
| alrg_w1_seasonal_ige | sIgE to Ambrosia artemisiifolia |
| ass_asthma_ctrl_baseline | Level of Asthma Control (controlled= 1, partly controlled= 2, uncontrolled= 3) |
| ass_asthma_sev_baseline | Asthma severity at baseline |
| cmb_ahi | AHI |
| cmb_allergic_rh1stis | Allergic rhinitis (yes=1, no=0) |
| cmb_atopic_dermatitis | Atopic dermatits (yes=1, no=0) |
| cmb_gerb_bin | GERB (1=positive, 0=negative) |
| cmb_ri_score | RI score (%) |
| gen_rs1042713 | rs1042713 (ADRB2) |
| gen_rs17576 | rs17576 (MMP9) |
| gen_rs1876828 | rs1876828 (CRHR1) |
| gen_rs242941 | rs242941 (CRHR1) |
| gen_rs37973 | rs37973 (GLCCI1) |
| gen_rs9910408 | rs9910408 (TBX21) |
| meas_fen0_baseline | FENO (ppb) |
| meas_fev1_baseline | FEV1 (% of predicted) |
| meas_mef50_baseline | MEF50 (% of predicted) |
| phys_age | Age [y] |
| phys_age_baseline | Age at onset asthma [y] |
| phys_basophils | Basophils (%, relative blood count) |
| phys_bmi_perc | BMI percentiles |
| phys_disease_duration | Duration of disease [y] |
| phys_eosinophils_blood | Eosinophil count in blood (%) |
| phys_eosinophils_dunger | Eosinophils (absolute count) |
| phys_gender | Gender |
| phys_height | Height [cm] |
| phys_hscrp | hsCRP (mg/ L) |
| phys_iga | IgA [g/L] |
| phys_ige_total | Total IgE (kIU/I) |
| phys_igg | IgG [g/L] |
| phys_igm | IgM [g/L] |
| phys_monocytes | Monocytes (relative blood count, %) |
| phys_neutrophils_blood | Neutrophil count in blood (%) |
| phys_platelets | Platelets (x 10^9/L) |
| phys_weight | Weight [kg] |
| resp_asthmalev_disc_1st-2nd | Response to treatment according to D level of asthma control (1= good, 2= moderate, 3= poor) |
| resp_asthmalev_disc_2nd-3rd | Response to treatment according to D level of asthma control (1= good, 2= moderate, 3= poor) |
| resp_asthmalev_disc_baseline-1st | Response to treatment according to D level of asthma control (1= good, 2= moderate, 3= poor) |
| resp_asthmalev_disc_baseline-2nd | Response to treatment according to D level of asthma control (1= good, 2= moderate, 3= poor) |
| resp_asthmalev_disc_baseline-3rd | Response to treatment according to D level of asthma control (1= good, 2= moderate, 3= poor) |
| resp_fen0_disc_1st-2nd | Response to treatment according to D FENO score (1= good, 2= moderate, 3= poor) |
| resp_fen0_disc_2nd-3rd | Response to treatment according to D FENO score (1= good, 2= moderate, 3= poor) |
| resp_fen0_disc_baseline-1st | Response to treatment according to D FENO score (1= good, 2= moderate, 3= poor) |
| resp_fen0_disc_baseline-2nd | Response to treatment according to D FENO score (1= good, 2= moderate, 3= poor) |
| resp_fen0_disc_baseline-3rd | Response to treatment according to D FENO score (1= good, 2= moderate, 3= poor) |
| resp_fev1_disc_1st-2nd | Response to treatment according to D FEV1 (1= good, 2= moderate, 3= poor) |
| resp_fev1_disc_2nd-3rd | Response to treatment according to D FEV1 (1= good, 2= moderate, 3= poor) |
| resp_fev1_disc_baseline-1st | Response to treatment according to D FEV1 (1= good, 2= moderate, 3= poor) |
| resp_fev1_disc_baseline-2nd | Response to treatment according to D FEV1 (1= good, 2= moderate, 3= poor) |
| resp_fev1_disc_baseline-3rd | Response to treatment according to D FEV1 (1= good, 2= moderate, 3= poor) |
| resp_mef50_disc_1st-2nd | Response to treatment according to D MEF50 (1= good, 2= moderate, 3= poor) |
| resp_mef50_disc_2nd-3rd | Response to treatment according to D MEF50 (1= good, 2= moderate, 3= poor) |
| resp_mef50_disc_baseline-1st | Response to treatment according to D MEF50 (1= good, 2= moderate, 3= poor) |
| resp_mef50_disc_baseline-2nd | Response to treatment according to D MEF50 (1= good, 2= moderate, 3= poor) |
| resp_mef50_disc_baseline-3rd | Response to treatment according to D MEF50 (1= good, 2= moderate, 3= poor) |
| th_antileuk_1st | LTRA (mg) |
| th_antileuk_2nd | LTRA (mg) |
| th_antileuk_3rd | LTRA (mg) |
| th_ch_1st-2nd | Change of treatment dose or class 1st to 2nd control (1=increase/step up, 2=decrease/step down 0=no) |
| th_ch_2nd-3rd | Change of treatment dose or class 2nd control to 3rd control (1=increase/step up, 2=decrease/step down 0=no) |
| th_ch_baseline-1st | Change of treatment dose or class baseline to 1st (1=increase/step up, 2=decrease/step down 0=no) |
| th_ICS_1st-2nd | Daily dose of ICS (mcg) |
| th_ICS_2nd-3rd | ICS 2nd control to 3rd control (1=yes, 0=no) |
| th_ICS_baseline-1st | ICS baseline to 1st (1=yes, 0=no) |
| th_ics_dose_1st | Daily dose of ICS (mcg) |
| th_ics_dose_2nd | Daily dose of ICS (mcg) |
| th_ics_dose_3rd | Daily dose of ICS (mcg) |
| th_ICS_LABA_1st-2nd | ICS+LABA 1st to 2nd control (1=yes, 0=no) |
| th_ICS_LABA_2nd-3rd | ICS+LABA 2nd control to 3rd control (1=yes, 0=no) |
| th_ICS_LABA_baseline-1st | ICS+LABA baseline to 1st (1=yes, 0=no) |
| th_ICS_LTRA_LABA_1st-2nd | ICS+LTRA (+/-LABA) 1st to 2nd control (1=yes, 0=no) |
| th_ICS_LTRA_LABA_2nd-3rd | ICS+LTRA (+/-LABA) 2nd control to 3rd control (1=yes, 0=no) |
| th_laba_1st_formoterol | LABA dose (mcg) |
| th_laba_1st_salmeterol | LABA dose (mcg) |
| th_laba_2nd_formoterol | LABA dose (mcg) |
| th_laba_2nd_salemeterol | LABA dose (mcg) |
| th_laba_2nd_salmeterol | LABA dose (mcg) |
| th_laba_3rd_formoterol | LABA dose (mcg) |
| th_laba_3rd_salmeterol | LABA dose (mcg) |
| th_laba_dose_1st | LABA |
| th_laba_dose_2nd | LABA |
| th_laba_dose_3rd | LABA |
| th_LTRA_1st-2nd | LTRA 1st to 2nd control (1=yes, 0=no) |
| th_LTRA_2nd-3rd | LTRA 2nd control to 3rd control (1=yes, 0=no) |
| th_saba_1st | SABA (puffs per month) |
| th_saba_2nd | SABA (puffs per month) |
| th_saba_3rd | SABA (puffs per month) |
| th_steroid_1st_budesonide | Inhaled steroid (generic name) |
| th_steroid_1st_budezonid | Inhaled steroid (generic name) |
| th_steroid_1st_ciklesonide | Inhaled steroid (generic name) |
| th_steroid_1st_ciklezonid | Inhaled steroid (generic name) |
| th_steroid_1st_fluticasone | Inhaled steroid (generic name) |
| th_steroid_2nd_budesonide | Inhaled steroid (generic name) |
| th_steroid_2nd_ciklesonide | Inhaled steroid (generic name) |
| th_steroid_2nd_fluticasone | Inhaled steroid (generic name) |
| th_steroid_3rd_budesonide | Inhaled steroid (generic name) |
| th_steroid_3rd_ciklesonide | Inhaled steroid (generic name) |
| th_steroid_3rd_fluticasone | Inhaled steroid (generic name) |
| th_treat_1st | No treatment (no treatment=1, on treatment=0) |
| th_treat_2nd | No treatment (no treatment=1, on treatment=0) |
| th_treat_3rd | No treatment (no treatment=1, on treatment=0) |

Table S5. Feature importance for the decision tree classifier

| Feature | Feature Importance % |
| --- | --- |
| meas_mef50_baseline | 24.1 |
| th_saba_3rd | 17.1 |
| phys_hscrp | 8.3 |
| phys_iga | 6.8 |
| phys_height | 4.5 |
| th_ICS_LABA_baseline-1st | 4.5 |
| th_ICS_LABA_2nd-3rd | 4.3 |
| ass_asthma_ctrl_baseline | 3.2 |
| phys_eosinophils_dunger | 2.8 |
| th_ch_baseline-1st | 2.7 |
| phys_neutrophils_blood | 2.5 |
| th_antileuk_2nd | 2.5 |
| phys_ige_total | 2.5 |
| phys_bmi_perc | 2.4 |
| meas_fen0_baseline | 2.4 |
| phys_monocytes | 2.3 |
| phys_age | 2.2 |
| gen_rs9910408 | 2 |
| phys_eosinophils_blood | 1.7 |
| phys_platelets | 1.4 |

# Supplement literature

1. Reddel HK, Taylor DR, Bateman ED, Boulet L-P, Boushey HA, Busse WW, et al. An Official American Thoracic Society/European Respiratory Society Statement: Asthma Control and Exacerbations. Am J Respir Crit Care Med. 2009;180:59–99.

2. Bousquet J, Heinzerling L, Bachert C, Papadopoulos NG, Bousquet PJ, Burney PG, et al. Practical guide to skin prick tests in allergy to aeroallergens. Allergy. 2012;67:18–24.

3. Dweik RA, Boggs PB, Erzurum SC, Irvin CG, Leigh MW, Lundberg JO, et al. An Official ATS Clinical Practice Guideline: Interpretation of Exhaled Nitric Oxide Levels (F e _NO_ ) for Clinical Applications. Am J Respir Crit Care Med. 2011;184:602–15.

4. Navratil M, Plavec D, Dodig S, Jelcic Z, Nogalo B, Erceg D, et al. Markers of systemic and lung inflammation in childhood asthma. J Asthma. 2009;46:822–8.

5. Streets CG, DeMeester TR. Ambulatory 24-hour esophageal pH monitoring: why, when, and what to do. J Clin Gastroenterol. 2003;37:14–22.

6. Hrvatsko pedijatrijsko društvo. R, Sabol Z, Turkalj M, Nenadić N, Marušić I, Kučić D, et al. Paediatria Croatica : časopis Hrvatskoga pedijatrijskog društva i Hrvatskog društva za školsku i sveučilišnu medicinu za pedijatriju, adolescentnu medicinu i granične struke. Paediatr. Croat. Klinika za dječje bolesti; 2013.

7. McKinney W. Data Structures for Statistical Computing in Python. Proc 9th Python Sci Conf. 2010;1697900:51–6.

8. Van Der Walt S, Colbert SC, Varoquaux G. The NumPy array: A structure for efficient numerical computation. Comput Sci Eng. 2011;13:22–30.

9. Pedregosa F, Varoquaux G, Gramfort A, Michel V, Thirion B, Grisel O, et al. Scikit-learn: Machine Learning in Python. J Mach Learn Res. 2011;12:2825–30.

10. Waskom M, Botvinnik O, O’Kane D, Hobson P, Lukauskas S, Gemperline DC, et al. Mwaskom/Seaborn: V0.8.1 (September 2017). doi.org. 2017;

11. Hunter JD. Matplotlib: A 2D Graphics Environment. Comput Sci Eng. 2007;9:90–5.

12. Sheehan WJ, Phipatanakul W. Indoor allergen exposure and asthma outcomes. Curr. Opin. Pediatr. Lippincott Williams and Wilkins; 2016. p. 772–7.

13. Li J, Wang H, Chen Y, Zheng J, Wong GW, Zhong N. House dust mite sensitization is the main risk factor for the increase in prevalence of wheeze in 13-14 year old schoolchildren in Guangzhou city, China. Clin Exp Allergy. 2013;43:n/a-n/a.

14. Lombardi C, Savi E, Ridolo E, Passalacqua G, Canonica GW. Is allergic sensitization relevant in severe asthma? Which allergens may be culprit? World Allergy Organ. J. BioMed Central Ltd.; 2017.
