# Supplementary figures and images for: Treatment outcome clustering patterns correspond to discrete asthma phenotypes in children
Source: Asthma Res Pract. 2021 Aug 3;7:11. doi: 10.1186/s40733-021-00077-x (PMC8330019; doi:10.1186/s40733-021-00077-x)

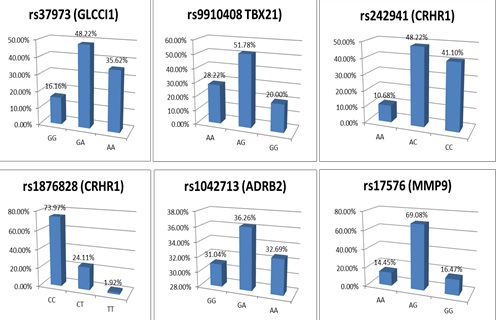

Supplement: Supplementary file 2 — Additional file 2. [file 40733_2021_77_MOESM2_ESM.jpg]
